# The impact of the COVID-19 pandemic on pharmacy personnel in primary care

**DOI:** 10.1017/S1463423622000445

**Published:** 2022-09-12

**Authors:** Natalie Weir, Rosemary Newham, Emma Dunlop, Aimee Ferguson, Marion Bennie

**Affiliations:** 1 Strathclyde Institute of Pharmacy and Biomedical Sciences, University of Strathclyde, 161 Cathedral Street, Glasgow G4 0RE, Scotland; 2 Public Health Scotland, Gyle Square, 1 South Gyle Crescent, Edinburgh EH12 9EB, Scotland

**Keywords:** COVID-19, general practice, job satisfaction, pharmacy practice, primary care

## Abstract

**Introduction:**

The coronavirus (COVID-19) pandemic has impacted healthcare worldwide. It has altered service delivery and posed challenges to practitioners in relation to workload, well-being and support. Within primary care, changes in physicians’ activities have been identified and innovative work solutions implemented. However, evidence is lacking regarding the impact of the pandemic on pharmacy personnel who work in primary care.

**Aim:**

To explore the impact of the pandemic on the working practice (including the type of services provided) and job satisfaction of pharmacists and pharmacy technicians within Scottish general practice. Due to the stressful nature of the pandemic, we hypothesise that job satisfaction will have been negatively affected.

**Methods:**

An online questionnaire was distributed in May–July 2021, approximately 15 months since initial lockdown measures in the UK. The questionnaire was informed by previous literature and underwent expert review and piloting. Analysis involved descriptive statistics, non-parametric statistical tests and thematic analysis.

**Results:**

180 participants responded (approximated 16.1% response rate): 134 pharmacists (74.4%) and 46 technicians (25.6%). Responses indicated greater involvement with administrative tasks and a reduction in the provision of clinical services, which was negatively perceived by pharmacists. There was an increase in remote working, although most participants continued to have a physical presence within general practices. Face-to-face interactions with patients reduced, which was negatively perceived by participants, and telephone consults were considered efficient yet less effective. Professional development activities were challenged by increased workloads and reduced support available. Although workplace stress was apparent, there was no indication of widespread job dissatisfaction.

**Conclusion:**

The pandemic has impacted pharmacists and technicians, but it is unknown if changes will be permanent, and there is a need to understand which changes should continue. Future research should explore the impact of altered service delivery, including remote working, on patient care.

## Introduction

The coronavirus (COVID-19) was first identified in December 2019 and progressed to a global pandemic in March 2020 (World Health Organization, 2020a). The pandemic caused a dramatic shift in healthcare services worldwide as healthcare systems prioritised the treatment of COVID-19 (Moynihan et al., 2021). Healthcare workers rapidly changed roles and their daily routines to mitigate disruption whilst reducing COVID-19 transmission risk (Chudasama et al., 2020; Adam et al., 2021). Nations have reported disruption of routine healthcare affecting services in 90% of countries (World Health Organization, 2020b) including hypertension management, cancer treatment and rehabilitation services (World Health Organization, 2020c; Chudasama et al., 2020), with a predicted ongoing increase in non-COVID-19 mortality as a result (World Health Organization, 2020c).

During the pandemic, there was prioritisation of COVID-19 in hospitals and primary care practitioners were at the forefront of dealing with the pandemic within the community (Jovičić-Bata et al., 2021; Lasalvia et al., 2021). Within general practice, innovative work solutions have been implemented to cope with the pressures of the pandemic, including remote patient consultations and telephone triage systems (The Lancet Respiratory, 2020; Khan et al., 2020; Verity et al., 2020; Mughal et al., 2021; Smyrnakis et al., 2021; Wanat et al., 2021) which offered general practitioners (GPs) the flexibility to work from home (Khan et al., 2020). This was considered an opportunity to improve the work-life balance of GPs and aid recruitment; however, an increase in workload and work-related stress have been reported (Khan et al., 2020; Cebrián-Cuenca et al., 2021; Wanat et al., 2021). Additionally, the lack of physical examination in remote consultations elicited anxiety as GPs were concerned about missing essential information for diagnoses (Wanat et al., 2021). Concerns have also been raised regarding professional isolation due to reduced patient and colleague interaction, alongside implications for professional development due to difficulties offering teaching and training within remote working environments (Khan et al., 2020).

Within primary care pharmacy, there is only limited research examining the impact of the pandemic on this workforce. Malson (2020) reflected that general practice pharmacists may have adopted remote working practices and virtual consultations in line with GPs (Malson, 2020). A qualitative study by Paudyal et al identified that general practice pharmacists have reduced provision of pharmacist-led specialist clinics, medication reviews and medicines reconciliation since the pandemic (Paudyal et al., 2021), yet these data were derived from only two participating general practice pharmacists. Additionally, a UK-wide survey conducted in Sept/Oct 2020 identified that the risk of leaving the profession was highest for pharmacists working in general practice (Royal Pharmaceutical Society, 2020), yet it is unclear if this is associated with the pandemic.

Within hospital and community pharmacy, there is growing evidence of the pandemic’s impact on these pharmacy personnel. An international review by Visacri et al. (2021) identified that service delivery had been altered within these sectors, with the community pharmacy sector in particular challenged by increasing workloads and prescriptions (Jovičić-Bata et al., 2021; Thorakkattil et al., 2021). Alterations in working practice in community and hospital sectors reduced contact with patients and increased telehealth communication (Adam et al., 2021; Koster et al., 2021; Parajuli et al., 2021; Thorakkattil et al., 2021), alongside the adoption of remote working (Bourdin et al., 2021). Additionally, pharmacists within these sectors have experienced increased stress and pressure (Imeri et al., 2021; McCallum et al., 2021) and reduced support for professional development since the pandemic (Imeri et al., 2021).

### Scottish context

**Figure 1.**
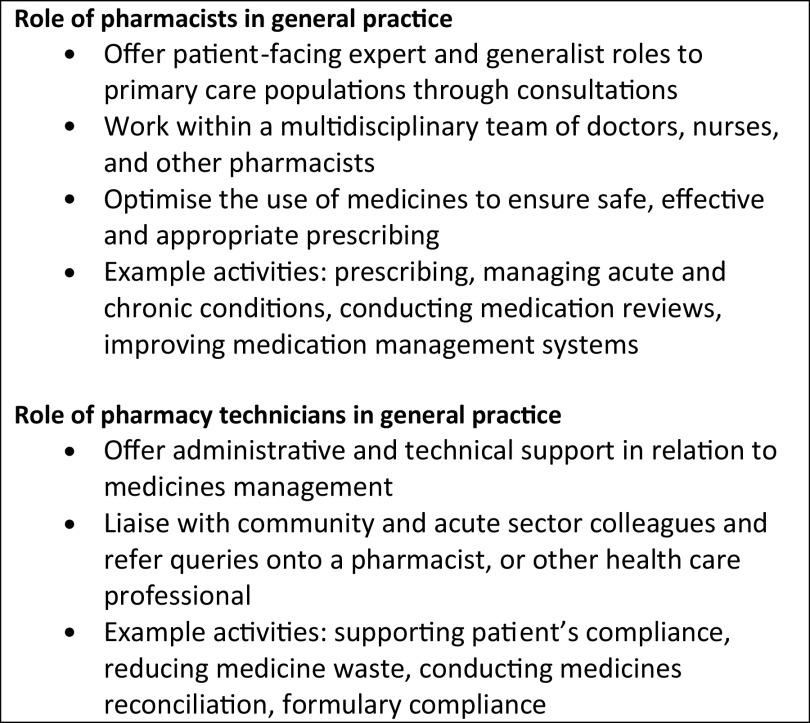
Role of pharmacists and pharmacy technicians within general practice (Royal Pharmaceutical Society, 2021; Claire et al., 2021; The Scottish Government, 2017)

The role of pharmacists and pharmacy technicians within general practice is varied and includes a range of clinical and administrative activities (e.g. medication reviews, medication reconciliation) whilst working within a multidisciplinary primary care environment (Figure 1) (Claire et al., 2021). Typically, pharmacists and pharmacy technicians increase capacity within primary care by addressing medication-related problems and support the management of long-term conditions (Levene et al., 2020; Claire et al., 2021; The Scottish Government, 2017a). Although there are similarities across community pharmacy, general practice pharmacy and hospital pharmacy within Scotland and the UK, some differences are apparent (Royal Pharmaceutical Society, 2021a; The Scottish Government, 2017a). The role of community pharmacy involves medication supply to the general population, offering targeted clinical services and being easily accessible to offer advice for acute and minor ailments (The Scottish Government, 2017a). General practice pharmacists and technicians instead situate within a general practice premise, and their role tends to involve formal medication reviews and supporting the long-term management of patient’s chronic conditions (The Scottish Government, 2017a). The hospital pharmacy sector has a role of supplying medicines to in-patients, and clinical services are delivered alongside doctors, nursing staff and other healthcare professionals (The Scottish Government, 2017a). Within Scotland, community and hospital pharmacy sectors are well established, yet the primary care pharmacy workforce is in development with £20.4m invested since 2015 to integrate pharmacy personnel in general practice (The Scottish Government, 2017b). This led to the introduction of the pharmacotherapy service in 2018, designed to enhance the clinical role of pharmacy personnel whilst relieving GPs of certain duties. The service is stratified into three level of tasks defined as core, advanced and specialist (see Appendix 1 for descriptions of the 17 tasks) (The Scottish Government, 2018). Increasingly, pharmacy technicians focus on core tasks with support from pharmacists as needed, whilst pharmacists have an additional focus on advanced and specialist tasks (Stewart et al., 2020).

To understand the pandemic’s impact on general practice pharmacy personnel, this study explores how the working practice (e.g. the type and delivery of services provided) and the job satisfaction of pharmacists and pharmacy technicians changed during the first 15 months of the pandemic. Due to the stressful nature of the COVID-19 pandemic (Imeri et al., 2021; McCallum et al., 2021), we also hypothesise that job satisfaction will have been negatively affected by the pandemic.

## Methods

An online questionnaire, hosted on Qualtrics© (version 2021), was used to ascertain participants’ perceptions. The study was conducted between May and June 2021. Ethical approval was granted by the Strathclyde Institute of Pharmacy and Biomedical Sciences Ethics Committee.

### Sample

Eligible participants were pharmacists and pharmacy technicians who worked within a general practice setting in Scotland in May 2021. This included those who commenced employment before and after the pandemic was announced in March 2020. The potential sample was estimated to be 1119 staff, comprising 851 pharmacists (WTE 698.5) and 268 pharmacy technicians (WTE 236.9) (NHS Education for Scotland, 2020).

### Participant recruitment and data collection

Representatives of the Scottish Practice Pharmacy and Prescribing Advisors Association (SP_3_AA) group and NHS Education for Scotland (NES) Education and Training Leads were sent an email with a link to the online questionnaire on Wednesday 19^th^ of May 2021. These individuals are pharmacists who have a managerial or educational role for pharmacists and technicians who work in general practice within the different geographical regions of Scotland. These pharmacists were instructed to disseminate the online questionnaire by forwarding the email with the questionnaire link to their email lists of pharmacist and technicians who work within general practice. A response deadline was set for the 4th of June 2021, with the email asked to be re-disseminated on 26th and 31st of May to act as a reminder and support dissemination to those who may have been on leave or who work part time. A Participant Information Sheet preceded the questionnaire, and participants provided informed consent.

### Questionnaire development

Overall, the questionnaire focused on: working tasks; interaction with patients; work setting and job satisfaction. Questionnaire items on working practice were informed by Stewart et al. (2020) and adapted to reflect changes instigated by the pandemic (e.g. remote working practices) and the newly implemented pharmacotherapy service (Scottish Pharmacy Practice and Prescribing Advisers Association, 2018). Participants were asked question to identify if, since the pandemic, they had increased, decreased, or not changed their time spent on each pharmacotherapy service task since the pandemic. Hassell et al.’s (2007) questionnaire measuring UK pharmacists’ job satisfaction was adapted for use. It is a validated satisfaction scale (Warr et al., 1979) and considered valid for pharmacists and technicians working within general practice (K Hassell, personal communication, 3rd of March 2021). Minor edits were made to ensure applicable language. The questionnaire comprised closed-ended questions with nominal, ordinal and Likert response options, which were interspersed with four open-ended questions focusing on the impact of the pandemic on service delivery, working practice, professional development and additional comments. Demographic characteristics were collected such as age, gender, job role and when participants commenced employment within general practice.

### Questionnaire review and piloting

Expert review of the questionnaire was conducted by the SP_3_AA group and NES, comprising pharmacists (*n* = 6) and technicians (*n* = 2), who commented on its appropriateness and relevance. Usability testing was conducted with broader research group members (*n* = 6). Piloting was conducted by an additional cohort of pharmacists (*n* = 6) and technicians (*n* = 2) who offered improvements in clarity and indicated time-to-complete. Pilot participants suggested improvements to the Likert scale. A 7-point Likert scale from ‘Extremely Dissatisfied’ to ‘Extremely Satisfied’ with equal positive and negative responses was selected. Piloting indicated the questionnaire took approximately 20 min to complete.

### Data management and analysis

Questionnaire responses obtained from Qualtrics© were exported into Microsoft Excel©, IBM Statistical Package for the Social Sciences (SPSS)© and NVivo© qualitative data analysis software.

#### Quantitative data analysis

Responses to closed-ended questions underwent descriptive statistics and presented either as modes, medians with interquartile range (IQR), total counts and percentages. Non-parametric tests were used to test the hypothesis that there was a reduction in participant’s job satisfaction. The Wilcoxon Matched-Pair Signed-Rank test was applied when the distribution of the differences between participants’ responses pre- and post-pandemic was symmetrical, with the Paired-Samples Sign Test applied when this assumption was not met. Statistical significance was determined using the Holm–Bonferroni method to account for multiple tests by correcting for Family-Wise Error Rate, with an overall type 1 error rate of α = 0.05 (Holm, 1979; Sinclair et al., 2013). The Mann–Whitney test was used to calculate if there were differences in overall job satisfaction between those newly employed and those employed pre-pandemic.

#### Qualitative data analysis

Responses to the open-ended questions were analysed within NVivo©. The data were structured using the framework method, whereby a matrix of summarised data is formed of codes (Gale et al., 2013). A coding framework was developed by NW based on 20% of participant’s responses. Overarching framework headings were based on the sections of the questionnaire and NW conducted coding inductively within these headings by assigning a paraphrase which represented the textual data. The coding framework was validated by NW, RN and AF by independently applying it to the analysis of 10% of responses. NW and ED applied the validated framework to 10% of data to ensure coding consistency. Thereafter, coding was conducted by NW and ED, with the coding framework iteratively adapted throughout ongoing discussions. A thematic analysis was conducted by NW making connections between codes (Gale et al., 2013), which was validated by ED by reading the themes and ensuring they were valid. The point of integration of the qualitative and quantitative data was at the interpretation and reporting level, where a ‘weaving approach’ was adopted with the results written as an integrative account (Fetters et al., 2013).

### Research team and reflexivity

This study was conducted by NW (Research Associate), RN (Research Fellow), AF (PhD Candidate), ED (Research Associate) and MB (Professor of Pharmacy) who each have experience of conducting questionnaires. Due to the anonymity of the participants, there were no concerns that established relationships between the researchers and participants would affect the results. NW, RN, AF, ED and MB work as researchers or academic staff members. MB also works as a Chief Pharmacist for Public Health Scotland, and NW also works as a community pharmacist. Therefore, NW may have biases/assumptions as a practicing pharmacist, yet this would likely be minimal as they have no work experience in general practice.

## Results

### Demographic characteristics

A total of 180 participants responded, of which 134 were pharmacists (74.4%) and 46 were pharmacy technicians (25.6%). This equates to an approximated 15.8% and 17.2% response rate for pharmacists and technicians, respectively. In total, 160 participants offered responses to the open-ended, free text questions which underwent qualitative analysis. Of the 180 respondents, a total of 151 participants (83.9%) were employed within general practice prior to the pandemic: 114 of which were pharmacists, and 37 were pharmacy technicians. A total of 29 participants (16.1%) were employed to work within general practice during the pandemic: 9 of which were technicians, and 20 were pharmacists. Full demographic characteristics are presented in Appendix 2. Representatives of 12 of the 14 healthcare regions in Scotland participated, with no representation from the two most rural regions (Orkney and the Western Isles). The majority of participants had >10 years professional experience; however, most had 1–5 years’ experience specifically in the general practice setting.

### Working tasks

Pharmacotherapy service tasks undertaken were explored to understand changes in work activity to ascertain if participants had increased, decreased or not changed the time spent on each task since the pandemic. Out of the 17 pharmacotherapy service tasks (defined in Appendix 1), Table 1 presents the eight tasks whereby at least a quarter of pharmacist (25%) either increased or decreased their time spent on these activities, with full data presented in Appendix 3. For the core tasks, there was most commonly an increase in activity observed, with over half of pharmacists increasing their time spent on Repeat Prescription Requests. For the advanced and specialist tasks, approximately a quarter of pharmacists decreased their time delivering Medication and Polypharmacy Reviews.

**Table 1.** Pharmacotherapy service tasks whereby at least 25% of pharmacists (*n* = 114) reported an increase or decrease in their time spent on these activities since the pandemic

Pharmacotherapy services	*n* (%) pharmacists who increased time spent on activity		*n* (%) pharmacists who reported no change in time spent on activity		*n* (%) pharmacists who decreased time spent on activity	
	*n*	%	*n*	%	*n*	%
Core tasks						
- Medicines reconciliation	40	35.1%	63	55.3%	11	9.6%
- Repeat prescription requests	59	51.8%	44	38.6%	11	9.6%
- Serial prescriptions	41	36.0%	66	57.9%	7	6.1%
- Hospital immediate discharge letters (IDLs)	42	36.8%	59	51.8%	13	11.4%
- Formulary adherence	7	6.1%	78	68.4%	29	25.4%
- Prescribing indicators and audits	6	5.3%	56	49.1%	52	45.6%
Advanced tasks						
- Medication review (more than 5 medicines)	18	15.8%	68	59.6%	28	24.6%
Specialist tasks						
- Polypharmacy reviews	17	14.9%	66	57.9%	31	27.2%

Table 2 presents the four core pharmacotherapy service tasks whereby at least 25% of technicians either increased or decreased their time on these activities, with full data presented in Appendix 4. Increased time spent on these activities was most commonly observed with the exception of the time spent on Prescribing Indicators and Audits.

**Table 2.** Pharmacotherapy service tasks whereby at least 25% of technicians (*n* = 37) reported an increase or decrease in their time on these activities since the pandemic

Pharmacotherapy services	Increased time spent on activity		No change in time spent on activity		Decreased time spent on activity	
	*n*	%	*n*	%	*n*	%
Core tasks						
- Medicines reconciliation	14	37.8%	18	48.6%	5	13.5%
- Serial prescriptions	11	29.7%	25	67.6%	1	2.7%
- Hospital immediate discharge letters (IDLs)	13	35.1%	19	51.4%	5	13.5%
- Prescribing indicators and audits	3	8.1%	22	59.5%	12	32.4%

#### Theme 1: Altered primary care landscape affecting service delivery

The changes highlighted in the quantitative data were elaborated upon within free-text responses. There was a perceived emphasis on offering core tasks, with a reduction in time spent on advanced and specialist clinical services offered to patients. This was most commonly negatively perceived by pharmacists as it can be ‘mundane and not clinically challenging’ (P69, Pharmacist). This change in service activity existed alongside a change in the focus of pharmacy’s role within primary care to support GPs and practices by freeing up their workload. Some participants believed that the development of primary care pharmacy services had been halted and felt undervalued:

*‘Other complex clinical work was deemed as adding to GP burden and not relieving it … GPs felt little value of the advanced level input and wanted only [core] and some [advanced] services … I felt unvalued after so many years of functioning at this advanced level’. (P71, Pharmacist)*

### Types of interaction

The way in which pharmacy personnel interacted was explored, with the most commonly reported response (i.e. the mode) presented in Table 3. Pre-pandemic, pharmacists reported some face-to-face interaction with patients. During the pandemic, modal responses indicated pharmacists and technicians most often reported no face-to-face and video interaction with patients during the pandemic. The modes for time spent conducting telephone communication reflected an increase for pharmacists and technicians, as had the time conducting work without direct patient interaction. Pharmacists employed during the pandemic reported less interaction via telephone (mode=21–40%), and technicians spent a notable amount of time conducting work without direct patient interaction (mode=61–80%).

**Table 3.** Types of interaction

Interaction	Pharmacists (*n* = 134) Mode % of time spent with each type of interaction			Technicians (*n* = 46) Mode % of time spent with each type of interaction		
	Employed before pandemic (*n* = 114)		Employed during pandemic (*n* = 20)	Employed before pandemic (*n* = 37)		Employed during pandemic (*n* = 9)
	Pre-pandemic	2021	2021	Pre-pandemic	2021	2021
Face-to-face patient interaction	1–20%	0%	0%	0%	0%	0%
Telephone patient interaction	1–20%	41–60%	21–40%	21–40%	61–80%	81–100%
Video patient interaction	0%	0%	0%	0%	0%	0%
Conducting work without direct patient interaction^*^	1–20%	21–40%	41–60%	41–60%	61–80%	61–80%

* For example looking at patient records/note-based review/engaging with other healthcare professionals without speaking to patients.

#### Theme 2: Efficiency and effectiveness of communication

Reduced face-to-face contact with patients was associated with a reliance on telephone communication due to remote working. Face-to-face interaction with patients was missed by pharmacists and technicians, who ‘found this part of the job extremely rewarding’ (P4, Pharmacy Technician). Telephone consultations were also considered efficient yet less effective as face-to-face communication, and some participants felt telephone consultations made it difficult to assess patients:

*‘I had to stop seeing patients in the practice altogether and manage my work via phone call … which is not ideal for every patient and did not allow me to carry out the examination I normally would and I was having to refer patients that I would normally have seen to my Advanced Nurse Practitioner/GP colleagues’. (P105, Pharmacist)*

### Work setting

The work setting was similar for both pharmacists and technicians. Overall, 95% of pharmacists and 87% of technicians reported they were working in general practice sites in 2021, a reduction from 98% and 92%, respectively, in 2020. However, for both pharmacists and technicians, 62% reported working at least partially from home during the pandemic, in comparison to approximately 3% in 2020. In 2021, one technician and no pharmacists reported on-site working within a care home, whereas in 2020 12.3% of pharmacists worked in a care home and 13.5% of technicians. Those who were newly employed during the pandemic reported similar working locations, yet were less commonly working remotely from home: 35.0% for pharmacists, and 33.3% for technicians. See Appendix 5 for full data on participants’ work setting.

#### Theme 3: Remote working: both a friend and foe

Participants had polarised opinions on remote working. Some participants found their work achievable when working remotely from home, and it had personal benefits such as an improvement in their work/life balance as it allowed for extra flexibility. The reduced time travelling to meetings or general practices was positively commented upon, as was the ease of scheduling and joining meetings remotely. For some, however, remote working was necessary due to social distancing requirements which limited space for pharmacy staff to work onsite in general practices. This impacted tasks which required the pharmacy staff to be onsite. For example, printing prescriptions required onsite staff to complete the task. Remote working also made it difficult to interact with colleagues which negatively impacted the working relationships as ‘all ad hoc conversations and learnings were removed’ (P43, Pharmacist Technicians). A prominent challenge was the limited ability of participants to be supervised, mentored and supported when working remotely:

*‘Due to practices not having space I have mostly been working from home. This means I have no available pharmacist for advice, guidance or overseeing my work. This means I have been able to do less due to safety’. (P20, Pharmacy Technician)*

### Job satisfaction

#### Job satisfaction: descriptive statistics

Overall job satisfaction was positively viewed, with median responses of > 5 (median scale from 1 to 7) for pharmacists and technicians before and during the pandemic. For participants employed before the pandemic, 52% of pharmacists and 73% of technicians reported either no change or an increase in their satisfaction for their job overall. A notable difference in the median score in relation to pharmacists’ satisfaction with patient contact was observed, reducing from a median of 5 (IQR = 4.00–6.00) pre-pandemic to a median of 3 (IQR = 2.00–5.00) in 2021. Descriptive statistics at an item and job role level are presented in Appendices 6–7.

#### Job satisfaction: statistical tests

For pharmacists employed before the pandemic, various determinants of overall satisfaction significantly reduced post-pandemic (see Table 4). This was most apparent with the level of patient contact pre and post pandemic (*P* < 0.001), with a corresponding difference in the median observed (as described above). For the other determinants, the pre- and post-pandemic median responses were ≥ 5. This suggested that, although there was a statistically significant reduction, there was not widespread dissatisfaction amongst participants. For technicians, there was no statistically significant change in participants reported satisfaction with any determinant of job satisfaction (Appendix 6), with all medians ≥ 4 indicating general satisfaction.

**Table 4.** Pharmacists’ job satisfaction for those employed before the pandemic (only significant results shown)

Determinants of job satisfaction	Pharmacists employed before pandemic (*n* = 114)						
	Pre-pandemic median (IQR)	May/June 2021 median (IQR)	Participants reporting reduction in satisfaction (*n*, %)	Participants reporting no change in satisfaction (*n*, %)	Participants reporting increase in satisfaction (*n*, %)	Test statistic	*P* value
Overall job satisfaction^*^	5 (5.00–6.00)	5 (3.00–5.00)	55 (48.2%)	46 (40.4%)	13 (11.4%)	-4.972	< 0.001^†^
Physical working conditions^*^	5 (4.00–6.00)	5 (4.00–6.00)	42 (36.8%)	55 (48.2%)	17 (14.9%)	-3.125	0.002^†^
Your colleagues and fellow workers^*^	6 (5.00–6.00)	6 (5.00–6.00)	27 (23.7%)	80 (70.2%)	7 (6.1%)	-3.258	0.001^†^
Recognition you get for good work^*^	5 (5.00–6.00)	5 (4.00–6.00)	36 (31.6%)	61 (53.5%)	17 (14.9%)	-3.060	0.002
Your salary^*^	5 (4.00–6.00)	5 (4.00–6.00)	17 (14.9%)	90 (78.9%)	7 (6.1%)	-2.315	0.021
Your hours of work^*^	6 (5.00–6.00)	6 (4.00–6.00)	30 (26.3%)	80 (70.2%)	4 (3.5%)	-4.287	< 0.001^†^
Amount of variety in your job^*^	5 (4.00–6.00)	5 (3.00–5.25)	49 (43.0%)	46 (40.4%)	19 (16.7%)	-4.088	< 0.001
Patient contact^*^	5 (4.00–6.00)	3 (2.00–5.00)	67 (58.8%)	38 (33.3%)	9 (7.9%)	-6.538	< 0.001^†^

KEY: 1 = extremely dissatisfied, 2 = very dissatisfied, 3 = somewhat dissatisfied, 4 = neutral, 5 = somewhat satisfied, 6 = very satisfied, 7 = extremely satisfied.

* Variables with a statistically significant reduction (corrected α < 0.05) in reported levels of satisfaction for pharmacists employed pre-pandemic (n = 114).

† Paired-Samples Sign Test conducted as distribution of the differences between participants responses pre- and post-pandemic was asymmetrical. Specific *P* values denoting significance were determined using the Holm–Bonferroni method (37, 38).

A comparison was made of overall satisfaction for participants employed during the pandemic to those employed pre-pandemic (Appendices 6 and 7). The median for overall job satisfaction was greater for those employed during the pandemic for pharmacists (median = 6, IQR = 4.25–6.00) and technicians (median = 6, IQR = 4.00–6.50). A Mann–Whitney U test indicated this difference was statistically significant for pharmacists (U test statistic = 8.213, *P* = 0.009) indicating greater satisfaction for those employed during the pandemic, but no statistical significance was identified for technicians (U test statistic = 2.448, *P* = 0.234).

#### Theme 4: Workplace stress

The qualitative findings identified that workplace stress was apparent. The pandemic and altered working practices were reported to increase the pressure felt by participants whilst doing their job. The changing role increased the workload for many participants and reduced the available time they had within their working day for other activities. Increased workloads posed challenges for undertaking professional development activities, with protected learning time no longer available to many participants. Additionally, concerns were raised regarding insufficient staffing to meet the increased workload, with some respondents highlighting the risk of patients receiving sub-optimal services:

*‘The volume of work has massively increased … I have felt rushed often to the point where I am trying to achieve tasks and get through the most amount of patients and I wonder if patients are receiving the same quality of care they were previously’. (P162, Pharmacist).*

## Discussion

This study explored the impact of the COVID-19 pandemic on the working practice and job satisfaction of pharmacists and pharmacy technicians working within Scottish general practice. It was conducted 15 months since the initial lockdown measures in March 2020, offering insight into the changes experienced by this workforce. Results indicated increased involvement in administrative medication management tasks such as medicines reconciliation. The related reduction in clinical services offered to patients was negatively perceived by pharmacists. The majority of participants continued to have a physical presence within general practice, but there was a notable increase in remote working and a reduction in face-to-face contact with patients. Patient-facing contact was missed by participants, and telephone consults were considered less effective than in-person consults. Qualitative findings identified that engagement with professional development activities was challenging during the pandemic due to increased workloads and lack of opportunity for support and mentoring. However, although workplace stress was apparent, there was not widespread dissatisfaction with participants’ jobs overall.

The findings indicate a change in work activity with greater involvement in some core pharmacotherapy service tasks which are more administrative in nature. Increased involvement with prescription requests could be related to the public’s initial ‘panic ordering’ of repeat medicines (Watt G, Mullin A and Blane D, 2020) and increased need for pharmacists to support GPs with this. The reduction in care-home related activity is likely associated with the challenges Scottish care homes faced with COVID-19 transmission rates (Burton et al., 2021), as well as altered care home policies in relation to who was able to enter the premises. This has been identified throughout the UK, where support for care homes by GPs has also been compromised due to remote working (Park *et al*., 2020). Changes in activity may also be explained by a shift in focus to supporting GPs to free up their workload, particularly as the majority of patients with COVID-19 are managed by GPs in the UK (Gray and Sanders, 2020). This could explain pharmacy personnel’s increased time spent on administrative tasks which offer immediate time-saving benefits, alongside reduced involvement with tasks which may be considered longer-term improvement work (e.g. formulary adherence). Paudyal et al similarly identified changes in the services provided by GP pharmacists (Paudyal et al., 2021), yet it remains unclear the impact of reduced provision of pharmacy services to primary care populations. This has clear implications for the continuity of patient care, and future research may wish to explore the impact of altered service delivery on pharmaceutical care outcomes (Weir et al., 2021). It is also unknown if such COVID-related changes to work practice will continue indefinitely and ongoing research to monitor this is needed, particularly considering the stress and negative associations with reduced patient contact.

This study identified an increase in pharmacists’ and technicians’ remote working, alongside reduced patient interaction and an increased reliance on telephone consultations with patients. This corroborates previous reflection that general practice pharmacists may have adopted remote working practices and virtual consultations (Malson, 2020) and mirrors findings of a UK wide survey indicating an increase in healthcare telephone consults (Horton et al.). Overall, these findings indicate that the pandemic has impacted primary care work processes, particularly in terms of the way in which patients are engaged with. Participants of the present study were less satisfied with their contact with patients since the pandemic and the effectiveness of telephone consults was questioned. This reflects previous findings that telephone consultations are convenient yet not always appropriate for patients who are new to a practice (Malson, 2020), on multiple medicines or with multiple co-morbidities (Hewitt et al., 2010; Malson, 2020), require a physical examination (Malson, 2020), have hearing impairments (Malson, 2020) or do not have access to a telephone or phone ‘credit’ (Verity et al., 2020). Previous research has also identified challenges building rapport during remote consultations (Verity et al., 2020) and identified that healthcare professionals are less likely to elicit additional concerns using this mode of communication (Hewitt et al., 2010). The sparse use of video consults may be considered surprising, as pre-pandemic there were extensive efforts to develop and subsequently adopt ‘Attend Anywhere’ (Wherton and Greenhalgh, 2020; Beattie et al., 2020), a video consultations platform which has national licence throughout Scotland. It is unclear why this technology was not adopted by pharmacy personnel in primary care despite its availability, and future work should explore patient and pharmacy personnel perceptions and preferences of remote consults in a post-COVID era (Murphy et al., 2021), which should inform evidenced-based guidelines on when, and how, to conduct remote consultations.

Despite some challenges experienced, there was not widespread dissatisfaction with participants’ job overall. Pharmacists and technicians employed during the pandemic were more satisfied when compared to those employed pre-pandemic. The reason for this difference is unclear, but it may be related to the fact that they were less likely to be working remotely from home. The findings are in contrast with results of a UK-wide survey conducted in Sept/Oct 2020 which identified that the risk of leaving the profession was highest for pharmacists working in general practice (Royal Pharmaceutical Society, 2020). This contrast could be explained by the different time points of data collection, as this study was conducted 15 months since the initial lockdown and some pandemic-related stressors may have alleviated, but it should also be acknowledged that the previous survey was UK wide and had only 83 general practice respondents. Overall, although widespread concerns with job satisfaction were not identified, if the challenges with workload identified continue indefinitely, it is possible that workplace satisfaction may be affected, and future research may be needed to monitor this.

The ability to engage with professional development activities appeared to be impacted by different facets of the pandemic including reduced protected learning time for pharmacists and limited support and mentoring, with professional development challenges also experienced by GPs within the UK and pharmacists in other sectors (Khan et al., 2020; Imeri et al., 2021). Considering this alongside the reduced provision of certain advanced and specialist services, it suggests that the professional and clinical remit of pharmacy personnel in Scottish general practice may not be currently progressing. This does not align with the Scottish pharmaceutical strategy whereby the skills of pharmacy personnel were envisaged to continually develop to deliver more complex pharmaceutical care to patients (The Scottish Government, 2017a). Additionally, the Royal Pharmaceutical Society’s 2030 strategic vision for general practice pharmacy proposes that the primary care pharmacy workforce should have a leading role in prescribing and managing long-term conditions (Royal Pharmaceutical Society, 2021b). Overall, the findings suggest the pandemic may have halted the professional progression of pharmacy personnel within primary care.

### Strengths and limitations

A strength of this study is the application of Hassell et al.’s valid and reliable questionnaire to explore job satisfaction, which offers reassurance regarding the validity of the results (Hassell et al., 2007). Other elements of the questionnaire were developed from previously published work and were adapted by considering advancements within primary care pharmacy in Scotland (Stewart et al., 2020). A strength of this paper is the mixed method approach. Often, the qualitative findings helped to confirm the quantitative findings and vice versa. Additionally, in many instances the qualitative findings have helped expand upon quantitative findings and illuminated participants thoughts and feeling surrounding changes in practice, which facilitate the development of conclusions and next step recommendations of this work. The questionnaire permitted responses from pharmacists and pharmacy technicians, offering a glimpse into how the pandemic has affected the pharmacy workforce in primary care. However, neither pharmacy support workers nor administrative assistants were sampled and it remains unknown how their role has been affected. A limitation is the low response rate and the potential for participation bias, and it is possible that this impacts the generalisability of the results, yet the extent of which is unclear. For example, it could be postulated that only participants with sufficient free time in their working day may have completed the questionnaire, which could impact the generalisability of the results relating to working tasks. Alternatively, it could be suggested that those most motivated to participate could be those with the most negative experiences of the pandemic’s impact on working practices, which could impact the generalisability of the job satisfaction results. As there is no central email distribution list of all potentially eligible participants, questionnaire dissemination was reliant on an email communication cascade directed by representatives of the SP_3_AA group and NES. It is therefore possible that some eligible participants may not have received the email comprising the questionnaire link, and it is possible that the email may not have been forwarded in a timely manner by all representatives. This was mitigated by repeated dissemination of the questionnaire and through close working with NHS management networks via the SP_3_AA group and NES Education and Training leads.

## Conclusions

Overall, the study identified that the pandemic has impacted pharmacists and pharmacy technicians working practice and has hindered professional development opportunities, yet job satisfaction remained adequate. The findings suggest that the pandemic may have negatively impacted the professional progression of pharmacy personnel within primary care. There is a need to monitor pharmacists’ and technicians’ work activities to understand if changes are transient or permanent. Ongoing exploration of barriers to professional development and workplace satisfaction will help to identify if the pharmacy workforce is engaged and content as the challenges of the pandemic subside. It is possible that the pharmaceutical care of patients has been impacted due to limited delivery of specialised clinical services, and future work may be needed to explore the effects of altered service delivery on clinical, humanistic, economic and service outcomes (Weir et al., 2021). Lastly, there is a need to understand which elements of remote working should continue, such as telephone consults, and research involving patients would help strategists understand if a blended primary care model mixing remote consults and face-to-face interaction is desirable.

## Supplementary material

For supplementary material accompanying this paper visit http://doi.org/10.1017/S1463423622000445. click here to view supplementary material
