# Supplementary material for: The impact of the COVID-19 pandemic on pharmacy personnel in primary care
Source: Prim Health Care Res Dev. 2022 Sep 12;23:e56. doi: 10.1017/S1463423622000445 (PMC9472301; doi:10.1017/S1463423622000445)
Supplement: Supplementary file 1 [file S1463423622000445sup001.zip › S1463423622000445sup001.docx]

Appendix 1: Pharmacotherapy service definitions – adapted from SP3AA (2019) [1]

| **Pharmacotherapy service levels** | **Pharmacotherapy service tasks** | **Definitions** |
| --- | --- | --- |
| **Core tasks:** | - Medicines reconciliation | Medicines reconciliation is the process by which an accurate and up-to-date list of medicines is obtained, documenting any discrepancies, changes, additions or deletions. It is performed any time care transfers across interfaces, (e.g. discharge from hospital, outpatient appointments). |
|  | - Acute and repeat prescribing requests | Prescribing requests which could be authorised / actioned could include:   - Hospital outpatient requests - Ad-hoc requests from patients / carers / other healthcare professionals - Review and reauthorisation of repeat prescriptions |
|  | - Serial prescriptions | Authorising, actioning and reviewing serial prescriptions. A serial prescription is for patients who are stabilised on their medication, for whom a long term prescription is generated which lasts for 24, 48 or 56 weeks. This prescription is dispensed by the community pharmacy at intervals specified by the GP practice (e.g. every 8 weeks). |
|  | - Hospital Immediate Discharge Letters (IDLs) | Undertake medicines reconciliation of Immediate Discharge Letters (IDLs) and update medication records. This may involve a review of medication, a discussion with patient/carer, or onward referral. |
|  | - Medicine safety reviews / recalls | Actioning medicines safety recalls and reviews including MHRA Drug Alerts and MHRA Drug Safety Updates. |
|  | - Monitoring high risk medicines | Monitoring high risk medicines, including:   - combinations of medicines associated with increased risk of harm e.g. “triple whammy” - single medicines requiring enhanced monitoring (e.g. DMARDs, lithium, warfarin) - single medicines associated with increased risk in specific clinical situations (e.g. DOACs, gabapentinoids in renal impairment).   This may involve screening of results against a set criteria of monitoring frequency, with possible escalation to pharmacist or GP. |
|  | - Non-clinical medication review (NCMR) | A technical review (notes based) of a list of a patient’s medication. The purpose is to identify and address simple issues such as housekeeping, and to highlight clinical issues for review by a clinician e.g. GP, pharmacist. |
|  | - Monitoring clinics | Clinics to provide support to patients/carers on medication related issue, such as:   - Review of compliance and concordance - Managing complex medication regimes or changes to medication e.g. antidepressant or antiepileptic cross titrations - Medication review at the request of patients/carers or following referral from other healthcare professionals - Targeted medication reviews (<5 medicines) - Management and monitoring of simple medication changes within competence (e.g. up-titration of angiotensin converting enzyme inhibitors [ACEIs])   This may involve onward referral to specialist pharmacist clinics or GP. |
|  | - Medication compliance reviews (patient’s own home) | Reviews include:   - Identification of deliberate non-compliance i.e. choice and non-deliberate e.g. cognitive impairment or physical factors - Provision of support to aid concordance e.g. reminder charts, physical aids, multicompartment devices, referral for medicines administration by community nurses or other carers - Referral for pharmacist led medication review |
|  | - Medication management advice and reviews (care homes) | Medicines management support to care homes includes review of and support to develop systems and processes for managing the ordering and supply of medicines; synchronisation and dose optimisation; non clinical medication review; medicines reconciliation. May involve referral for pharmacist led medication review |
|  | - Formulary adherence | Promoting formulary adherence is integral to all aspects of the pharmacotherapy service. |
|  | - Prescribing indicators and audits | Review of prescribing indicator reports such as NTIs, local prescribing indicators, PRISMs data. May involve provision of prescribing data to practices; audits of prescribing as agreed with the GP practice and/or in line with local priorities. |
| **Advanced tasks:** | - Medication review (more than 5 medicines) | Optimise use of medicines for a patient and identify medicines no longer indicated/required, appropriate dose changes or new medicines to address unmet need(s). Note-based review with changes being discussed with patient or carer. |
|  | - Resolving high risk medicine problems | The resolution of clinical issues arising from the ‘Monitoring high risk medicines’ Level 1 task. |
| **Specialist tasks:** | - Polypharmacy reviews | Medication review with patient and/or carer. Can involve prescribing, monitoring or recommending therapy adjustments. Targets highest risk patient populations (e.g. frail patients, frequent hospital admission). |
|  | - Telephone triage | Triaging and signposting medication-related requests (e.g. special requests, patient enquiries) via telephone or at a reception desk. This may result in referral to other health care professionals or services. |
|  | - Specialist clinics | Comprehensive management of complex patients and their condition, where the pharmacist will manage the patient and their condition. |

[1] SP3AA (2019). National Pharmacotherapy Service Specification Version 3
