# Supplementary material for: The impact of the COVID-19 pandemic on pharmacy personnel in primary care
Source: Prim Health Care Res Dev. 2022 Sep 12;23:e56. doi: 10.1017/S1463423622000445 (PMC9472301; doi:10.1017/S1463423622000445)
Supplement: Supplementary file 1 [file S1463423622000445sup001.zip › S1463423622000445sup002.docx]

Appendix 2: Demographic characteristics of participants (n=180)

| **Demographic characteristics** | **All participants n=180** n (%) | **Pharmacists n=134** n (%) | **Technicians n=46** n (%) |
| --- | --- | --- | --- |
| **Gender** | | | |
| Male | 30 (16.7) | 26 (19.4) | 4 (8.7) |
| Female | 145 (80.6) | 103 (76.9) | 42 (91.3) |
| Nonbinary | 1 (0.6) | 1 (0.7) | 0 (0.0) |
| Prefer not to say | 4 (2.2) | 4 (3.0) | 0 (0.0) |
| **Length of time qualified (years)** | | | |
| <1 | 3 (1.7) | 1 (0.7) | 2 (4.3) |
| 1-5 | 25 (13.9) | 16 (11.9) | 9 (19.6) |
| 6-9 | 37 (20.6) | 30 (22.4) | 7 (15.2) |
| 10-14 | 32 (17.8) | 24 (17.9) | 8 (17.4) |
| 15-19 | 28 (15.6) | 20 (14.9) | 8 (17.4) |
| 20+ | 55 (30.6) | 43 (32.1) | 12 (26.1) |
| **Length of experience in GP setting** | | | |
| <1 | 20 (11.1) | 12 (9.0) | 8 (17.4) |
| 1-5 | 112 (62.2) | 85 (63.4) | 27 (58.7) |
| 6-9 | 20 (11.1) | 12 (9.0) | 8 (17.4) |
| 10-14 | 15 (8.3) | 13 (9.7) | 2 (4.3) |
| 15-19 | 5 (2.8) | 4 (3.0) | 1 (2.2) |
| 20+ | 8 (4.4) | 8 (6.0) | 0 |
