# Supplementary material for: The impact of the COVID-19 pandemic on pharmacy personnel in primary care
Source: Prim Health Care Res Dev. 2022 Sep 12;23:e56. doi: 10.1017/S1463423622000445 (PMC9472301; doi:10.1017/S1463423622000445)
Supplement: Supplementary file 1 [file S1463423622000445sup001.zip › S1463423622000445sup003.docx]

Appendix 3: Percentage (%) of pharmacist (n=114) reporting changes in time spent on all pharmacotherapy service tasks since pandemic

| **Pharmacotherapy Services** | **Increased time spent on activity** | | **No change in time spent on activity** | | | **Decreased time spent on activity** | |  |
| --- | --- | --- | --- | --- | --- | --- | --- | --- |
|  | **n** | **%** | **n** | **%** | **n** | | **%** |  |
| **Core** | | | | | | | | |
| Medicines Reconciliation | 40 | 35.1% | 63 | 55.3% | 11 | | 9.6% |  |
| Repeat Prescribing Requests | 59 | 51.8% | 44 | 38.6% | 11 | | 9.6% |  |
| Serial Prescriptions | 41 | 36.0% | 66 | 57.9% | 7 | | 6.1% |  |
| Hospital Immediate Discharge Letters (IDLs) | 42 | 36.8% | 59 | 51.8% | 13 | | 11.4% |  |
| Medicine Safety Reviews / Recalls | 5 | 4.4% | 88 | 77.2% | 21 | | 18.4% |  |
| Monitoring High Risk Medicines | 21 | 18.4% | 89 | 78.1% | 4 | | 3.5% |  |
| Non-Clinical Medication Review (NCMR) | 7 | 6.1% | 99 | 86.8% | 8 | | 7.0% |  |
| Monitoring Clinics | 8 | 7.0% | 95 | 83.3% | 11 | | 9.6% |  |
| Medication Compliance Review (Patient’s Own Home) | 1 | 0.9% | 90 | 78.9% | 23 | | 20.2% |  |
| Medication Management Advice and Reviews (Care Homes) | 5 | 4.4% | 84 | 73.7% | 25 | | 21.9% |  |
| Formulary Adherence | 7 | 6.1% | 78 | 68.4% | 29 | | 25.4% |  |
| Prescribing Indicators and Audits | 6 | 5.3% | 56 | 49.1% | 52 | | 45.6% |  |
| **Advanced** | | | | | | | | |
| Medication Review (more than 5 medicines) | 18 | 15.8% | 68 | 59.6% | 28 | | 24.6% |  |
| Resolving High Risk Medicine Problems | 14 | 12.3% | 89 | 78.1% | 11 | | 9.6% |  |
| **Specialist** | | | | | | | | |
| Polypharmacy Reviews | 17 | 14.9% | 66 | 57.9% | 31 | | 27.2% |  |
| Telephone Triage | 23 | 20.2% | 88 | 77.2% | 3 | | 2.6% |  |
| Specialist Clinics | 17 | 14.9% | 78 | 68.4% | 19 | | 16.7% |  |
