# Supplementary material for: The impact of the COVID-19 pandemic on pharmacy personnel in primary care
Source: Prim Health Care Res Dev. 2022 Sep 12;23:e56. doi: 10.1017/S1463423622000445 (PMC9472301; doi:10.1017/S1463423622000445)
Supplement: Supplementary file 1 [file S1463423622000445sup001.zip › S1463423622000445sup004.docx]

Appendix 4: Percentage (%) of technicians (n=37) reporting changes in time spent on each pharmacotherapy service tasks since pandemic

| **Pharmacotherapy Services** | **Increased time spent on activity** | | **No change in time spent on activity** | | **Decreased time spent on activity** | |
| --- | --- | --- | --- | --- | --- | --- |
|  | n | % | n | % | n | % |
| **Core** | | | | | | |
| Medicines Reconciliation | 14 | 37.8% | 18 | 48.6% | 5 | 13.5% |
| Repeat Prescribing Requests | 6 | 16.2% | 28 | 75.7% | 3 | 8.1% |
| Serial Prescriptions | 11 | 29.7% | 25 | 67.6% | 1 | 2.7% |
| Hospital Immediate Discharge Letters (IDLs) | 13 | 35.1% | 19 | 51.4% | 5 | 13.5% |
| Medicine Safety Reviews / Recalls | 4 | 10.8% | 29 | 78.4% | 4 | 10.8% |
| Monitoring High Risk Medicines | 2 | 5.4% | 31 | 83.8% | 4 | 10.8% |
| Non-Clinical Medication Review (NCMR) | 4 | 10.8% | 27 | 73.0% | 6 | 16.2% |
| Monitoring Clinics | 0 | 0.0% | 36 | 97.3% | 1 | 2.7% |
| Medication Compliance Review (Patient’s Own Home) | 1 | 2.7% | 28 | 75.7% | 8 | 21.6% |
| Medication Management Advice and Reviews (Care Homes) | 6 | 16.2% | 27 | 73.0% | 4 | 10.8% |
| Formulary Adherence | 3 | 8.1% | 27 | 73.0% | 7 | 18.9% |
| Prescribing Indicators and Audits | 3 | 8.1% | 22 | 59.5% | 12 | 32.4% |
