# Supplementary material for: The impact of the COVID-19 pandemic on pharmacy personnel in primary care
Source: Prim Health Care Res Dev. 2022 Sep 12;23:e56. doi: 10.1017/S1463423622000445 (PMC9472301; doi:10.1017/S1463423622000445)
Supplement: Supplementary file 1 [file S1463423622000445sup001.zip › S1463423622000445sup005.docx]

Appendix 5: Pharmacists’ and technicians’ work setting

Figure A. Pharmacists’ work setting

*i.e. a pharmacy office not attached to a GP surgery

Figure B. Technicians’ work setting

*i.e. a pharmacy office not attached to a GP surgery
