# Supplementary material for: The impact of the COVID-19 pandemic on pharmacy personnel in primary care
Source: Prim Health Care Res Dev. 2022 Sep 12;23:e56. doi: 10.1017/S1463423622000445 (PMC9472301; doi:10.1017/S1463423622000445)
Supplement: Supplementary file 1 [file S1463423622000445sup001.zip › S1463423622000445sup006.docx]

Appendix 6: Full data on technicians’ job satisfaction

| **Determinants of job satisfaction** | **TECHNICIANS (n=46)** | | | | | | | | |
| --- | --- | --- | --- | --- | --- | --- | --- | --- | --- |
|  | **Technicians employed  before pandemic (n=37)** | | | | | | | | **Employed during pandemic (n=9)** |
|  | **PRE-PANDEMIC**  median (IQR) | **MAY/JUNE 2021**  median (IQR) | **PARTICIPANTS REPORTING REDUCTION IN SATISFACTION** (n, %) | **PARTICIPANTS REPORTING  NO CHANGE IN SATISFACTION** (n, %) | **PARTICIPANTS REPORTING INCREASE IN SATISFACTION** (n,% ) | **test  statistic** | **p value** | **MAY/JUNE  2021**  median  (IQR) | |
| Overall job satisfaction | 6  (5.00-6.00) | 5  (4.00-6.00) | 10  (27.0%) | 23  (62.2%) | 4  (10.8%) | -1.336 | 0.18† | 6  (4.00-6.500) | |
| Physical working conditions | 6  (4.00-6.00) | 5  (3.00-6.00) | 13  (35.1%) | 17  (45.9%) | 7  (18.9%) | -1.118 | 0.263† | 5  (4.00-6.00) | |
| Freedom to choose your own method of working | 5  (4.00-6.00) | 5  (3.50-6.00) | 10  (27.0%) | 16  (43.2%) | 11  (29.7%) | -0.228 | 0.820 | 4  (4.00-6.00) | |
| Your colleagues and fellow workers | 6  (5.00-6.00) | 6  (4.00-6.00) | 12  (32.4%) | 20  (54.1%) | 5  (13.5%) | -1.455 | 0.143† | 6  (4.50-7.00) | |
| Recognition you get for good work | 5  (4.00-6.00) | 5  (4.00-6.00) | 8  (21.6%) | 20  (54.1%) | 9  (24.3%) | 0.000 | 1.000† | 5  (4.50-6.50) | |
| Amount of responsibility you are given | 5  (5.00-6.00) | 5  (4.50-6.00) | 9  (24.3%) | 19  (51.4%) | 9  (24.3%) | 0.000 | 1.000† | 5  (5.00-6.00) | |
| Your salary | 5  (4.00-6.00) | 5  (3.00-6.00) | 9  (24.3%) | 26  (70.3%) | 2  (5.4%) | -1.809 | 0.065† | 6  (4.00-6.00) | |
| Opportunity to use your abilities | 5  (4.00-6.00) | 5  (3.00-6.00) | 9  (24.3%) | 22  (59.5%) | 6  (16.2%) | -0.516 | 0.607† | 5  (4.00-6.00) | |
| Your hours of work | 5  (4.00-6.00) | 5  (4.00-6.00) | 5  (13.5%) | 29  (78.4%) | 3  (8.1%) | -0.354 | 0.727† | 6  (4.50-6.50) | |
| Amount of variety in your job | 5  (3.50-6.00) | 5  (3.00-5.50) | 12  (32.4%) | 18  (48.6%) | 7  (18.9%) | -0.918 | 0.359† | 5  (3.50-6.00) | |
| Patient contact | 5  (4.00-6.00) | 4  (2.00-5.00) | 12  (32.4%) | 23  (62.2%) | 2  (5.4%) | -2.405 | 0.013† | 4  (3.00-6.00) | |

***KEY: 1 = Extremely Dissatisfied, 2 = Very Dissatisfied, 3 = Somewhat Dissatisfied, 4 = Neutral, 5 = Somewhat Satisfied, 6 = Very Satisfied, 7 = Extremely Satisfied***

*†Paired-Samples Sign Test conducted as distribution of the differences between participants responses pre- and post- pandemic was asymmetrical*
