# Supplementary material for: The impact of the COVID-19 pandemic on pharmacy personnel in primary care
Source: Prim Health Care Res Dev. 2022 Sep 12;23:e56. doi: 10.1017/S1463423622000445 (PMC9472301; doi:10.1017/S1463423622000445)
Supplement: Supplementary file 1 [file S1463423622000445sup001.zip › S1463423622000445sup007.docx]

Appendix 7: Full data on pharmacists’ job satisfaction

| **Determinants of job satisfaction** | **PHARMACISTS (n=134)** | | | | | | | | | |
| --- | --- | --- | --- | --- | --- | --- | --- | --- | --- | --- |
|  | **Employed before  pandemic (n=114)** | | | | | | | | **Employed during pandemic (n=20)** | |
|  | **PRE-PANDEMIC** median (IQR) | **MAY/JUNE 2021**  median (IQR) | **PARTICIPANTS REPORTING REDUCTION IN SATISFACTION** (n, %) | **PARTICIPANTS REPORTING  NO CHANGE IN SATISFACTION** (n, %) | **PARTICIPANTS REPORTING INCREASE IN SATISFACTION** (n,% ) | **test  statistic** | **p value** | **MAY/JUNE 2021**  median  (IQR) | |  |
| Overall job satisfaction | 5  (5.00-6.00) | 5  (3.00-5.00) | 55  (48.2%) | 46  (40.4%) | 13  (11.4%) | -4.972 | <0.001*† | 6  (4.25-6.00) | |  |
| Physical working conditions | 5  (4.00-6.00) | 5  (4.00-6.00) | 42 (36.8%) | 55  (48.2%) | 17 (14.9%) | -3.125 | 0.002*† | 5.5  (3.25-6.75) | |  |
| Freedom to choose your own method of working | 5  (4.00-6.00) | 4  (3.00-6.00) | 39  (34.2%) | 49  (43.0%) | 26  (22.8%) | -2.000 | 0.045 | 5  (4.00-6.00) | |  |
| Your colleagues and fellow workers | 6  (5.00-6.00) | 6  (5.00-6.00) | 27 (23.7%) | 80 (70.2%) | 7 (6.1%) | -3.258 | 0.001*† | 6  (6.00-6.00) | |  |
| Recognition you get for good work | 5  (5.00-6.00) | 5  (4.00-6.00) | 36 (31.6%) | 61  (53.5%) | 17 (14.9%) | -3.060 | 0.002* | 5  (4.25-6.00) | |  |
| Amount of responsibility you are given | 5.5 (5.00-6.00) | 5  (5.00-6.00) | 25  (21.9%) | 68 (59.6%) | 21 (18.4%) | -0.442 | 0.658† | 5  (4.25-6.00) | |  |
| Your salary | 5  (4.00-6.00) | 5  (4.00-6.00) | 17  (14.9%) | 90  (78.9%) | 7  (6.1%) | -2.315 | 0.021* | 5  (3.00-5.00) | |  |
| Opportunity to use your abilities | 5  (4.00-6.00) | 5  (3.00-6.00) | 45  (39.5%) | 50 (43.9%) | 19  (16.7%) | -3.125 | 0.020† | 5  (3.50-6.00) | |  |
| Your hours of work | 6  (5.00-6.00) | 6  (4.00-6.00) | 30 (26.3%) | 80 (70.2%) | 4  (3.5%) | -4.287 | <0.001*† | 6  (5.00-6.00) | |  |
| Amount of variety in your job | 5  (4.00-6.00) | 5  (3.00-5.25) | 49 (43.0%) | 46 (40.4%) | 19  (16.7%) | -4.088 | <0.001* | 5  (5.00-6.00) | |  |
| Patient contact | 5  (4.00-6.00) | 3  (2.00-5.00) | 67  (58.8%) | 38  (33.3%) | 9  (7.9%) | -6.538 | <0.001*† | 5  (3.00-5.00) | |  |

***KEY: 1 = Extremely Dissatisfied, 2 = Very Dissatisfied, 3 = Somewhat Dissatisfied, 4 = Neutral, 5 = Somewhat Satisfied, 6 = Very Satisfied, 7 = Extremely Satisfied*****Variables with a statistically significant reduction (corrected α < 0.05) in reported levels of satisfaction for pharmacists employed pre-pandemic (n=114)
†Paired-Samples Sign Test conducted as distribution of the differences between participants responses pre- and post- pandemic was asymmetrical*
